# Playful approach to Hansen’s disease through an educational comic book

**DOI:** 10.1590/0034-7167-2024-0464

**Published:** 2026-08-03

**Authors:** Denise Quandt, Carine Vendruscolo, Ivana Griboggi, Antônio Francisco Jacó Rodrigues, Clodis Maria Tavares, Daiana Kloh Khalaf

**Affiliations:** I Universidade Federal do Paraná. Curitiba, Paraná, Brazil; II Universidade do Estado de Santa Catarina. Florianópolis, Santa Catarina, Brazil; III Universidade Federal de Alagoas. Maceió, Alagoas, Brazil

**Keywords:** Leprosy, Graphic Novel, Nursing, Education Continuing, Primary Health Care., Lepra, Historietas, Enfermería, Educación Continua, Atención Primaria de Salud.

## Abstract

**Objectives:**

to develop a creative educational technology in the form of an educational comic book on Hansen’s disease for primary health care professionals.

**Methods:**

an applied qualitative study focused on the development of an educational technology, using a multiprofessional approach.

**Results:**

the development of the educational comic book encompassed the following aspects: the importance of scientific research; the role of the Brazilian Unified Health System and primary health care; stigma; signs and symptoms; diagnosis; classification; and supervised treatment. The comic book, organized into eight chapters, highlights the relevance of health education and the multidimensional impact of Hansen’s disease, seeking to mitigate stigma and improve patient care.

**Final Considerations:**

the educational comic book underscores the need for an integrated approach to Hansen’s disease management, promoting empathy, understanding, and ongoing efforts to combat stigma and improve the quality of life of people affected by the disease.

## RESUMEN

**Objetivos:**

desarrollar una tecnología educativa creativa en formato de cómic sobre la enfermedad de Hansen, dirigida a profesionales de la Atención Primaria de Salud.

**Métodos:**

estudio cualitativo aplicado al desarrollo de una tecnología educativa, con un enfoque multiprofesional.

**Resultados:**

el diseño del cómic abordó los siguientes aspectos: importancia de la investigación científica; papel del Sistema Único de Salud de Brasil y de la Atención Primaria de Salud; estigma; sintomatología; diagnóstico; clasificación; y tratamiento supervisado. La obra se compone de ocho capítulos y destaca la relevancia de la educación sanitaria, así como el impacto multidimensional de la enfermedad de Hansen, para mitigar el estigma y mejorar la atención a los pacientes.

**Consideraciones Finales:**

el cómic subraya la necesidad de un enfoque integrado en el manejo de la enfermedad de Hansen, promoviendo empatía, comprensión y esfuerzos continuos para combatir el estigma y mejorar la calidad de vida de las personas afectadas.

## INTRODUCTION

Despite technological advances in health care, Hansen’s disease (leprosy) remains a significant challenge in developing countries^(1-3)^. It continues as an unfinished narrative, with roots that go back to biblical times; it has been studied for centuries, yet it is still poorly understood in the health sciences^(4-6)^. Classified as a neglected tropical disease (NTD) caused by *Mycobacterium leprae*, this slowly progressing condition predominantly manifests in the skin and peripheral nerves^(7,8)^.

However, Hansen’s disease transcends biological and treatment-related aspects: it also involves social, psychological, and cultural dimensions related to stigma. Deformities and disabilities resulting from the disease have a profound impact on people affected by the disease, creating a reality marked by stigmatization and social exclusion^(9-11)^. Characteristics such as its slow progression, symptoms that can be mistaken for other conditions, and the risk of disability demand a differentiated approach, particularly for prevention, early detection, treatment, and the care provided to these individuals^(9-12)^.

In this sense, the process of becoming ill is influenced not only by individual and collective biological characteristics but also by economic production and historical, social, and cultural developments, as well as by advances in technical and scientific knowledge to which humanity has been exposed; in this context, social inequality is a key determinant of diseases such as Hansen’s disease^(13)^.

Amid these complexities, vulnerabilities, and challenges related to this disease as a public health problem, the Brazilian Unified Health System (Sistema Único de Saúde - SUS) and its health care network (Rede de Atenção à Saúde - RAS) stand out. In this network, primary health care (PHC), considered the organizing hub of the system, is essential to ensure comprehensive care by providing surveillance, prevention, diagnosis, treatment, and rehabilitation actions^(14-16)^.

Within PHC, nurses play a key role in caring for people affected by Hansen’s disease. These professionals are positioned to implement actions at both the individual and collective levels, emphasizing comprehensive care and taking into account the context in which patients live^(17-19)^. However, to effectively fulfill this role and address Hansen’s disease, these professionals, as well as other members of the health care team, must develop specific knowledge and skills and adopt a proactive stance toward the early recognition of lesions.

National and international studies have pointed to a lack of preparedness among health professionals, especially regarding the early diagnosis of Hansen’s disease^(19-24)^. In light of this reality, there is a need to strengthen technical and scientific knowledge not only in clinical practice but also from undergraduate education onward, in order to enhance the effectiveness of actions and improve outcomes in professional practice.

Given this context, the following research question was formulated: “How can the technical and scientific knowledge of PHC professionals about Hansen’s disease be strengthened while the topic is addressed creatively?”. This article seeks to answer this question by presenting the development of an educational technology in the form of an educational comic book, which uses an engaging and effective approach to address the complexity of technical and scientific knowledge in the training of these professionals.

## OBJECTIVES

To develop a creative educational technology in the form of an educational comic book on Hansen’s disease for primary health care professionals.

## METHODS

### Ethical aspects

This study derives from a Master’s thesis in a professional master’s program in Nursing titled “Hansen’s disease in primary health care: a playful approach using comic books”. The study was exempted from ethical review and approval by a research ethics committee because it was based exclusively on publicly available data to support its theoretical foundation. For the same reason, patient consent was not required.

### Study design

This is a qualitative, exploratory, methodological study. The Analysis, Design, Development, Implementation, and Evaluation (ADDIE) model^(25)^ was used to guide the development of an educational technology in the form of an educational comic book^(24)^. The last phase of this model, Evaluation, is not addressed in this study. The study was carried out between October 2022 and November 2023 in Curitiba, Paraná, Brazil.

### Theoretical and methodological framework

Methodological research has been applied in nursing to develop measurement instruments; develop care, management, and educational technologies; translate and perform cross-cultural adaptation of instruments; and validate nursing diagnoses, outcomes, and interventions^(24-26)^.

In this study, methodological research supported the development of an educational technology, providing the steps required to achieve the proposed objective.

### Methodological procedures

The development of the educational comic book followed the four phases of the ADDIE model. The first phase, Analysis, involved conducting a literature review and document analysis. The second phase, Design, included selecting the content; structuring, revising, and adapting the script with a comic book writer; and choosing the characters. The third phase, Development, comprised layout design and artwork. The fourth phase, Implementation, was instrumental in disseminating and promoting the educational comic book.

### Study setting

The target audience was primary health care professionals, especially nurses, who provide first-contact care to people at different stages of life.

### Data collection and organization

The information and documents collected in the initial stage were organized in an Excel spreadsheet, which included the Brazilian state, type of document, title, year of publication, and web address. The findings helped define the most appropriate format for presenting the educational technology, taking into account the target audience, the needs to be met, and the context, which informed the study phases described below.

### Study phases

**Phase 1 - Analysis** - An in-depth exploration of the context of Hansen’s disease in Brazil was conducted to understand the complexity of the social determinants of health, stigma, and planned interventions to address and control the disease. This work involved document analysis of the websites of state health departments to identify guidelines, protocols, manuals, and recommendations related to Hansen’s disease control activities carried out by health professionals.

Ministerial, state, and international documents, as well as scientific articles, were consulted. Content was selected chronologically to guide readers through the disease, the origins of the associated stigma, the role of PHC, signs and symptoms, diagnosis, classification, treatment, and vulnerability.

In addition, a narrative literature review was conducted in BIREME and on the Coordination for the Improvement of Higher Education Personnel (*Coordenação de Aperfeiçoamento de Pessoal de Nível Superior* - CAPES) portal, using a standardized search strategy with the Portuguese keywords “*hanseníase*”, “*controle da hanseníase*”, and “*hanseníase e APS*”.

**Phase 2 - Design** - This phase required a multiprofessional approach involving the researchers, the cartoonist, and the comic book writer. For didactic purposes, it was subdivided into stages that co-occurred. The development of the educational comic book was organized into the following stages.

Script structuring stage - Based on the content selected in Phase 1 - Analysis, this stage adopted a playful approach, using everyday dialogues between health professionals and health service users to raise awareness and capture readers’ attention.

Script adaptation stage - After the researchers finalized the script, a comic book writer adapted and refined the text, bringing it to life.

Character creation stage - Character selection involved defining characteristics such as physical appearance, race/ethnicity, gender, profession, and social context so the characters would feel more like everyday people and the stories would evoke real people, settings, and day-to-day situations.

Drawing stage - Adobe Photoshop was used to create the artwork and design. Initial sketches were created based on the previously formatted script. Once approved, these sketches moved on to Phase 3, in which the details of each scene were finalized.

Hyperlink selection stage - To underpin the content defined in Phase 1 and promote interactivity in the educational comic book, links to additional reading materials on the topic presented in each chapter were added, when appropriate.

**Phase 3 - Development** - This phase was subdivided into stages to organize the workflow.

Layout design stage - This stage was carried out in collaboration with the cartoonist and the comic book writer. During this period, the number of panels and speech balloons per page was defined, as well as their size and iconic visual language, all organized from left to right^(27)^.

Coloring stage - The final construction stage involved adding color to the drawings and characters after all chapters of the story had been completed. With the cartoonist’s support, a color palette was selected for the panels and characters, bringing the stories to life.

Throughout all stages, special attention was paid to content presentation, language, and overall aesthetics, without compromising the development of the technical and scientific content related to the topic. This process also involved using the past tense and single spacing between lines in both the introductory section and the body of the educational comic book.

**Phase 4 - Implementation** - The main strategy was broad dissemination on leading open access nursing and health platforms, such as Cofenplay. In addition, a partnership was established with the Municipal Health Department of Curitiba to distribute the educational comic book in print form to primary health care units in the city.

Although it is an educational comic book with fictional characters, the narrative on Hansen’s disease in PHC was carefully developed. The aim was to portray the disease sensitively, avoiding stereotypes. The team also ensured clinical plausibility and respect for clinical characteristics, even in a fictional context. The approach included demystifying Hansen’s disease through clear messages, and character development respected cultural and ethnic diversity. The story underwent multiple rounds of review by the researchers to ensure an ethical and responsible approach.

## RESULTS

The script development was based on the content identified and updated in the narrative review and document analysis. The first draft of the narrative was created by outlining a playful story centered on two main characters: nurse Denise and a curious young man named Pedro.

**Figure 1:**
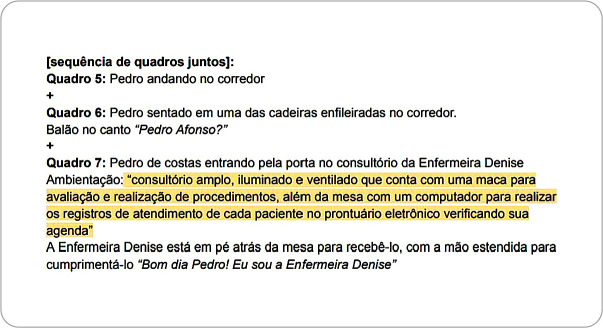
Adaptation of the script

A comic book writer then revised this script to give the story and characters greater pace and bring them to life. Figure 1 illustrates an example of the text after the writer’s revisions.

Once all stages were completed, the team decided to divide the educational comic book into eight chapters, presented below.

Chapter I. The Hansen’s disease enigma - Illustrates the curiosity of a young student who, motivated to conduct a research project on Hansen’s disease, faces numerous questions about the topic and struggles to carry out high-quality research, so he decides to seek help from a health professional.

This chapter underscores the importance of research and the search for scientific evidence to build knowledge, both in academic training and in professional development.

Chapter II. In search of knowledge: a pleasant surprise - Highlights the importance of the SUS and the services offered in PHC, portraying how care is organized within a health care unit and showing the importance of nurses as key actors in building knowledge and implementing health education.

**Figure 2:**
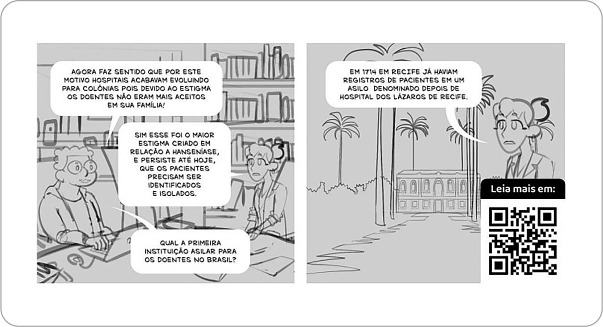
Layout of the chapter on the history of Hansen’s disease

Chapter III. Uncovering the history - The chapter aims to draw the reader’s attention to the history of Hansen’s disease and raise awareness of the psychosocial aspects associated with the disease, stigma, and its consequences, which have spanned centuries and persist to this day. These elements are depicted in Figure 2.

**Figure 3:**
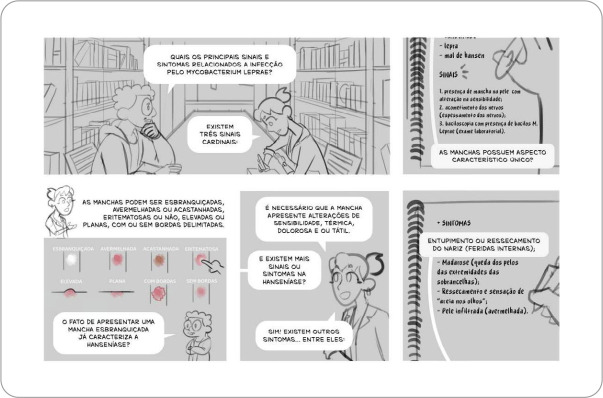
Layout of the chapter on signs and symptoms

Chapter IV. Exploring the disease: signs and symptoms - The central focus of this chapter is to clarify the signs and symptoms of Hansen’s disease, presenting the main manifestations and cardinal signs, and raising the issue of health professionals’ lack of confidence and preparedness to diagnose and care for people affected by this disease (Figure 3).

**Figure 4:**
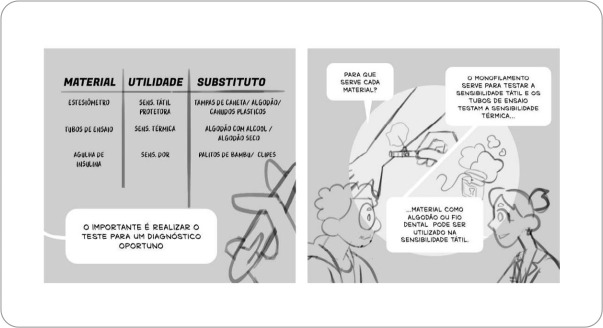
Layout of the chapter on diagnosis

Chapter V. Diagnosis - This chapter addresses a crucial aspect of care, diagnosis, which many authors identify as a critical weakness in the care of people with suspected Hansen’s disease. It clearly and simply presents the examinations and tests performed to confirm the diagnosis and provides guidance on the necessary materials and possible alternatives^(7)^ (Figure 4).

Chapter VI. Classification - This chapter clarifies and further explores the classification of Hansen’s disease, its clinical forms, and the main characteristics of each category in the Madrid classification^(28)^, namely: the paucibacillary form is subdivided into indeterminate and tuberculoid forms, both characterized by up to five skin lesions; the multibacillary form is subdivided into borderline and lepromatous forms, which generally present with more than five lesions. The clinical aspects and specific features of each form are presented playfully.

**Figure 5:**
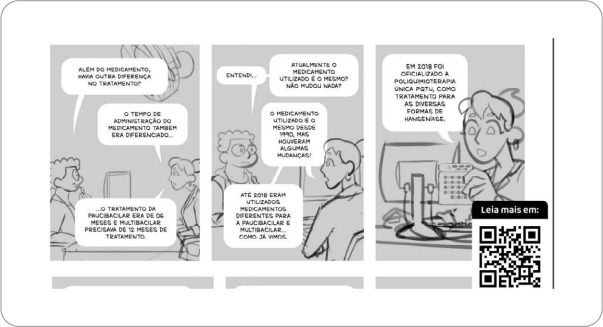
Layout of the chapter on treatment

Chapter VII. Treatment - This chapter traces the history and evolution of treatment, showing the importance of monthly supervised multidrug therapy (MDT) administered by nurses to achieve a cure. It provides guidance on the correct use and appropriate prescription for each form of the disease, in line with Ministry of Health guidelines. It also emphasizes ongoing follow-up throughout treatment to prevent treatment default and the mandatory notification of cases after the diagnosis is confirmed (Figure 5).

Chapter VIII. Vulnerability - The final chapter presents Hansen’s disease as a neglected tropical disease, linking it closely to issues of vulnerability. It also highlights the impact of this classification on the lives of people affected by the disease and underscores the importance of research aimed at reducing the effects of stigma.

## DISCUSSION

Permanent health education - PHE (*educação permanente em saúde* - EPS) is pivotal to addressing gaps in professional education and in the training of health professionals. Conceived as an educational policy, PHE seeks to address a lack of preparedness that can result from an education focused solely on the development of operational technical skills, without integrating multidisciplinary knowledge, including ethical, social, and political dimensions^(29,30)^.

In this sense, the development of the educational comic book reflects a need perceived in everyday health care practice, where professionals receive specific demands from the communities they serve, including people affected by Hansen’s disease, and are often unfamiliar with the care pathway recommended by the Ministry of Health for these cases. It is also important to emphasize the essential role of health professionals’ knowledge, expertise, skills, and proactivity-especially nurses, who are usually the first to care for people with the disease in PHC-in designing and implementing effective strategies for the early lesion recognition. These qualities strengthen efforts to address the disease and help pave the way for its eradication in Brazil^(11)^.

Thus, the use of educational comic books stands out as an effective approach because it transforms complex content into engaging and motivating learning activities, making reading and comprehension enjoyable, encouraging closer engagement with the topic, and promoting changes in the teaching and learning process^(27,31)^. Studies^(32,33)^ support this effectiveness, showing the potential of comic books as playful tools for active learning.

In this sense, Figures 4 and 5 illustrate the playful potential of comic books by addressing challenging content-such as the history of Hansen’s disease and its signs and symptoms-through engaging language closely aligned with professional practice. The literature highlights that using comic books in the classroom fosters reflection on new information and, consequently, contributes to knowledge gains in a way that complements the traditional formats adopted in educational institutions^(34)^.

The chapter exploring the history of Hansen’s disease and its stigma is designed to immerse readers in the historical context and lived experiences, encouraging deep reflection on the reality of social exclusion faced by people affected by the disease. This approach seeks to foster empathy while demystifying prejudice and stigma associated with this neglected disease in Brazil. It is essential to underscore the relevance of this educational strategy, since reducing stigma and prejudice is one of the core principles advocated by the World Health Organization (WHO) as a pathway toward eradicating Hansen’s disease^(2)^.

The inclusion of creativity in developing educational comic books is crucial for engaging and motivating professionals from the outset, as illustrated in “Chapter I. The Hansen’s disease enigma”. The engaging narrative, centered on a young student’s curiosity, is strategically crafted to capture attention and spark interest in the topic. By framing the storyline as an enigma, the comic book stirs readers’ curiosity and encourages active involvement in the search for answers. This approach not only makes learning more appealing but also creates an emotional connection, since identifying with the protagonist can foster a personal bond with the narrative.

Thus, creativity in developing educational comic books is not merely stylistic; it also serves as an effective pedagogical strategy, making learning engaging and memorable. This idea is consistent with PHE, which views the practice setting as one of the key spaces for developing responses to the needs of individuals and communities^(29)^.

The visual dimension of educational comic books is also essential, as it offers clarity and accessibility when presenting complex information in a simplified way. “Chapter IV. Exploring the disease: signs and symptoms” illustrates this strategy by focusing on the visual understanding of the fundamental aspects of Hansen’s disease^(35)^.

By using visual elements such as illustrations and diagrams, educational comic books provide a graphic representation of concepts, making the content more understandable and memorable. This approach not only addresses diverse learning styles, including those who benefit most from visual information, but also enhances teaching effectiveness, as visualization can simplify complex concepts. In this way, combining the visual approach of comic books with the exploration of the signs and symptoms of Hansen’s disease in Chapter IV highlights the effectiveness of this strategy for communicating health information in an accessible and educational manner.

By situating knowledge in a practical context, the educational comic book aims to make learning more applicable to nurses’ daily work in PHC settings. “Chapter V. Diagnosis” specifically illustrates this approach by providing information directly relevant to health professionals’ everyday practice. This strategy aims to establish a direct connection between the concepts presented in the comic book and the real situations encountered by nurses in PHC, fostering a deeper understanding and more effective application of the knowledge acquired.

This focus shows that health education is strengthened through meaningful learning, underscoring the importance of linking theoretical learning to professional practice. Thus, contextualizing knowledge within nurses’ daily routines (Chapter V) not only reinforces the relevance of the information presented but also helps prepare professionals to better face challenges in the health care field^(36)^.

The educational comic book seeks to foster reflection and empathy by addressing the history of Hansen’s disease and its stigma, promoting a deeper understanding of the experiences of people affected by the disease. It also aims to reinforce the urgency of adequately preparing health professionals who care for people affected by the disease so they can recognize lesions early and provide timely management. In “Chapter VII. Treatment”, the emphasis goes beyond technical aspects, explicitly highlighting the nurse’s compassionate role in supporting patients. In addition to acknowledging the importance of technical skills, this approach underscores the need to humanize care.

By shifting away from care centered solely on the biomedical model, the focus moves toward a holistic, comprehensive approach to caring for people affected by Hansen’s disease. This change in perspective reflects an understanding that treatment should not be limited to the physical aspects of the disease; it must also encompass psychosocial and emotional dimensions, acknowledging the complexity of patients’ experiences. Beyond these aspects, it is important to recognize that this is a neglected tropical disease and, when not detected early, a disabling one, which therefore requires concerted efforts from health professionals and health authorities to mitigate its impact^(37)^.

By including treatment in Chapter VII, the educational comic book provides information on medical practice while building a narrative that raises awareness among health professionals and readers of the importance of a compassionate, comprehensive approach to caring for people affected by Hansen’s disease.

This approach, aligned with contemporary health care trends, underscores the importance of a broader, patient-centered perspective to address this disease and its associated challenges.

The multisensory nature of educational comic books, which combine visual and textual elements, is intended to offer a rich and engaging learning experience. Figure 3, which illustrates “Chapter II. In search of knowledge: a pleasant surprise”, deepens understanding of nurses’ work environment in PHC. In this sense, creative tools for use in permanent health education, such as the comic books developed in this study, not only meet the need for ongoing professional development: they also transform the teaching and learning process into a more appealing and effective experience for adults in health care settings^(38)^.

Thus, educational comic books emerge as a promising pedagogical tool, capable of integrating diverse content in an accessible and engaging format. Their visual and narrative language helps create playful learning environments, promoting dialogue, knowledge exchange, and the strengthening of individuals’ social and imaginative dimensions^(35,39)^.

Despite these strengths, the use of educational comic books remains poorly established in educational settings, particularly in the health field, and further research is needed to demonstrate their effectiveness and applicability. Nevertheless, there is evidence of institutional efforts-especially by the Ministry of Health-to incorporate them into educational initiatives. This movement is justified by the ability of this resource to address a wide range of topics and reach different population groups, including those experiencing vulnerability, through inclusive and context-sensitive communication strategies^(35,38,39)^.

### Study limitations

The limitations of this study include the need to validate the educational comic book developed, establish its construct and face validity, and evaluate its impact within PHE. Product validation is a critical step to ensure the effectiveness and appropriateness of the educational material, confirming that it meets its proposed objectives. This process involves review and assessment by subject-matter experts, educators, if applicable, the target audience, to verify the quality, accuracy, and relevance of the comic book content.

Another limitation is the need to assess the impact of the material on PHE. Although educational comic books have the potential to be effective learning tools, it is essential to assess how these interventions affect the ongoing education of health professionals. Impact evaluation may include indicators such as changes in professionals’ knowledge, attitudes, and practices, as well as their perceptions of the usefulness and relevance of educational comic books in the context of PHE.

In addition, the generalizability of the findings is limited because the study was conducted in a specific setting and with a specific profile of health professionals. Replicating the study in different contexts and with more diverse samples is therefore recommended to validate the applicability and effectiveness of educational comic books as a PHE tool in various health-related situations and among different professional profiles.

Given the importance of validating and evaluating the educational material, the research team chose to analyze the impact of the educational comic book after its print dissemination, undertaken through a partnership between the Municipal Health Department and the Municipal Department of Education in a municipality in the Metropolitan Region of Curitiba.

The evaluation will be conducted using a structured questionnaire built in Google Forms, following a predefined script to assess the following aspects: design and clarity of the text, creativity and originality, educational effectiveness, and suitability for the target audience. The instrument will combine Likert-scale and open-ended questions to capture subjective aspects of each reader’s experience.

### Contributions to the field of nursing, health, and public policy

In this context, educational comic books emerge as a valuable tool that can not only stimulate learners’ creativity but also strengthen interactions among them. This perspective is supported by a recent study that examined the use of comic books in physical education to promote health in Brazilian elementary schools^(39)^.

Beyond their educational potential, comic books also serve other important purposes. The playful nature of these graphic narratives can significantly help reduce stress and anxiety levels among patients and health professionals. Reading comic books can be a valuable strategy to help health workers and the community cope with challenging experiences while fostering empathy through stories shared by others^(40,41)^.

Multiprofessional collaboration was essential to the development of this study, given the need to adapt the technical and scientific content proposed by the authors in the initial script to the material’s characteristics and the specific requirements of comic book design.

## FINAL CONSIDERATIONS

This study highlights the important contribution of educational comic books as an educational tool to strengthen professional education in health, particularly in the context of Hansen’s disease, which remains a neglected disease in Brazil. The findings show that their use not only enriches the teaching and learning process by making complex topics more accessible and engaging but also promotes learners’ creativity and strengthens social interactions.

In addition to demonstrating the potential of comic books to address educational aspects, the results also point to their contribution to overcoming emotional challenges by reducing stress and anxiety levels among patients and health professionals. At the same time, this tool fosters empathy through shared narratives, strengthening the technical and scientific knowledge of PHC professionals who care for people affected by Hansen’s disease while promoting a creative approach to the topic.

However, it is essential to acknowledge the study’s limitations. The findings are specific to a particular context, limiting their generalizability. Replicating the study in different settings and with more diverse samples is therefore necessary to validate the applicability and effectiveness of comic books as a tool for permanent health education in various health-related situations and among different professional profiles.

## Data Availability

The research data are available in a repository: https://doi.org/10.48331/scielodata.IT9VIP.
