# Supplementary material for: Playful approach to Hansen’s disease through an educational comic book
Source: Rev Bras Enferm. 2026 Aug 3;79:e20240464. doi: 10.1590/0034-7167-2024-0464 (PMC13435858; doi:10.1590/0034-7167-2024-0464)
Supplement: Translation

# Translation: Abordagem lúdica da Hanseníase por meio de histórias em quadrinhos

**DOI:** 10.1590/0034-7167-2024-0464pt

**Authors:** Denise Quandt, Carine Vendruscolo, Ivana Griboggi, Antônio Francisco Jacó Rodrigues, Clodis Maria Tavares, Daiana Kloh Khalaf

**Affiliations:** I Universidade Federal do Paraná. Curitiba, Paraná, Brasil; II Universidade do Estado de Santa Catarina. Florianópolis, Santa Catarina, Brasil; III Universidade Federal de Alagoas. Maceió, Alagoas, Brasil

**Keywords:** Hanseníase, História em Quadrinhos, Enfermagem, Educação Permanente, Atenção Primária à, Saúde.

## Abstract

**Objetivos:**

construir uma tecnologia educacional criativa na forma de história em quadrinhos sobre a hanseníase, para profissionais da Atenção Primária à Saúde.

**Métodos:**

pesquisa qualitativa aplicada em uma tecnologia educativa, usando uma abordagem multiprofissional.

**Resultados:**

a construção da história em quadrinhos abrangeu os aspectos: importância da pesquisa científica, papel do Sistema Único de Saúde e da Atenção Primária à Saúde, estigma, sinais e sintomas, diagnóstico, classificação e tratamento supervisionado. A obra conta com oito capítulos e destacou a relevância da educação em saúde bem como o impacto multidimensional da hanseníase, visando mitigar o estigma e melhorar o cuidado aos pacientes.

**Considerações Finais:**

a história em quadrinhos ressaltou a necessidade de uma abordagem integrada no manejo da hanseníase, promovendo empatia, compreensão e esforços contínuos para combater o estigma e melhorar a qualidade de vida dos afetados.

## INTRODUÇÃO

Apesar dos avanços tecnológicos na área da saúde, a hanseníase persiste como um desafio significativo em países em desenvolvimento^(1-3)^. Ela permanece como uma narrativa em aberto, com raízes que remontam aos tempos bíblicos; tem sido estudada ao longo dos séculos, mas ainda é mal compreendida pelas ciências da saúde^(4-6)^. Classificada como uma “doença tropical negligenciada” (DTN), causada pela *Mycobacterium leprae*, essa enfermidade de evolução lenta manifesta-se predominantemente na pele e nos nervos^(7,8)^.

Entretanto, a hanseníase transcende os aspectos biológicos e de tratamento: envolve também dimensões sociais, psicológicas e culturais relacionadas ao estigma. As deformidades e as incapacidades decorrentes da doença impactam profundamente a vida das pessoas afetadas, gerando uma realidade marcada pela estigmatização e exclusão social^(9-11)^. Peculiaridades como a evolução lenta, os sintomas confundíveis com outras enfermidades e o prognóstico de incapacidades demandam uma abordagem diferenciada, especialmente na prevenção, detecção precoce, tratamento e cuidados prestados a esses indivíduos^(9-12)^.

Nesse sentido, pode-se afirmar que o processo de adoecimento é influenciado não apenas pelas características biológicas individuais e coletivas, mas também pela produção econômica, pela evolução histórica, social e cultural, bem como pelos avanços do conhecimento técnico científico aos quais a humanidade tem sido exposta; em tal cenário, a desigualdade social constitui fator determinante para doenças como a hanseníase^(13)^.

Nesse quadro de complexidades, fragilidades e desafios relacionados a essa doença como problema de saúde pública, destaca-se o Sistema Único de Saúde (SUS), com sua Rede de Atenção à Saúde (RAS). A Atenção Primária à Saúde (APS), considerada a célula organizadora do sistema, é essencial para alcançar a integralidade dos cuidados, proporcionando ações de vigilância, prevenção, diagnóstico, tratamento e reabilitação^(14-16)^.

No âmbito da APS, os enfermeiros assumem um papel crucial no cuidado à pessoa com hanseníase. Esses profissionais têm o potencial de realizar ações em nível individual e coletivo, destacando a importância do atendimento com foco nos cuidados integrais e considerando o contexto em que o paciente está inserido^(17-19)^. Entretanto, para efetivamente desempenhar esse papel e combater a hanseníase, esses profissionais, assim como os demais membros da equipe de saúde, devem desenvolver conhecimentos, habilidades específicas e proatividade no reconhecimento precoce das lesões.

Estudos nacionais e internacionais têm apontado o despreparo dos profissionais de saúde, especialmente no tocante ao diagnóstico precoce da hanseníase^(19-24)^. Diante dessa realidade, surge a necessidade de fortalecer o conhecimento técnico científico, não apenas no campo da prática, mas também desde a graduação, visando elevar a eficiência das ações e melhorar os resultados obtidos na atuação profissional.

Considerando esse contexto, foi elaborada a seguinte questão de pesquisa: “Como fortalecer o conhecimento técnico-científico dos profissionais de saúde da APS para a atenção à hanseníase e, ao mesmo tempo, abordar a temática de maneira criativa?”. Este artigo propõe responder a essa questão por meio do desenvolvimento de uma tecnologia educacional baseada em histórias em quadrinhos (HQ), que utiliza uma abordagem leve e eficaz para trabalhar a complexidade do conhecimento técnico-científico na formação desses profissionais.

## OBJETIVOS

Construir uma tecnologia educacional criativa na forma de história em quadrinhos sobre a hanseníase, para profissionais da Atenção Primária à Saúde.

## MÉTODOS

### Aspectos éticos

Este estudo é resultado de uma pesquisa de mestrado profissional em enfermagem cujo título é “Hanseníase na Atenção Primária em Saúde: uma abordagem lúdica com histórias em quadrinhos”. A pesquisa foi dispensada da revisão ética e da aprovação por um comitê de ética, pois foi elaborada com base apenas em dados públicos, para sua fundamentação teórica. Pelo mesmo motivo, o consentimento do paciente não foi necessário.

### Tipo de estudo

Trata-se de um estudo qualitativo, exploratório e de caráter metodológico. Foi utilizado o modelo ADDIE (*Analysis, Design, Development, Implementation e Evaluation*)^(25)^, com o propósito de desenvolver uma tecnologia educativa na forma de história em quadrinhos, também conhecida como “gibi” no Brasil^(24)^. Ressalta-se que a última fase do referido modelo não será abordada neste estudo. A pesquisa transcorreu entre outubro de 2022 e novembro de 2023, na cidade de Curitiba, Paraná, Brasil.

### Referencial teórico-metodológico

A pesquisa metodológica tem sido aplicada dentro da área da enfermagem nas seguintes modalidades: elaboração de instrumentos de medida; elaboração de tecnologias assistenciais, gerenciais e/ou educacionais; translação e adaptação transcultural de instrumentos; e validação de diagnósticos, resultados e intervenções de enfermagem^(24-26)^.

O presente estudo utilizou-se desse tipo de pesquisa para o desenvolver a modalidade de elaboração de tecnologia educacional, já que subsidia as etapas necessárias para alcançar o objetivo proposto.

### Procedimentos metodológicos

O desenvolvimento da história em quadrinhos passou pelas quatro fases do modelo ADDIE. A primeira fase, denominada Análise, envolveu a revisão da literatura e a pesquisa documental. A segunda fase, chamada de Desenho, desdobrou-se na seleção do conteúdo; na estruturação, revisão e adequação do roteiro por um roteirista; e, por fim, na escolha dos personagens. Já a terceira fase, nomeada Desenvolvimento, compreendeu a diagramação e a confecção dos desenhos. A quarta fase, Implementação, desempenhou um papel fundamental na disseminação e na promoção da referida história em quadrinhos.

### Cenário do estudo

O público-alvo foram os profissionais da APS, em especial enfermeiros, por serem responsáveis pelo primeiro atendimento aos usuários em diferentes fases da vida.

### Coleta e organização dos dados

As informações e os documentos coletados na etapa inicial foram organizados em uma planilha do Excel, incluindo informações como identificação do Estado, tipo de documento, título, ano de publicação e endereço digital. Os resultados obtidos delimitaram o melhor formato de apresentação da tecnologia educativa, considerando o público-alvo, as necessidades a serem supridas e o contexto, o que orientou a realização das seguintes etapas do estudo.

### Etapas do trabalho

**Fase 1 - Análise** - Houve uma imersão aprofundada no contexto da hanseníase no Brasil, para compreender a complexidade dos determinantes sociais, os estigmas e as possíveis intervenções planejadas para o enfrentamento e controle da doença. Esse trabalho envolveu uma pesquisa documental nos sites das secretarias estaduais de saúde para identificar diretrizes, protocolos, manuais e orientações relacionados ao enfrentamento e controle da hanseníase por profissionais de saúde.

Foram consultados documentos ministeriais, estaduais e internacionais, bem como artigos científicos. A seleção de conteúdo ocorreu de maneira cronológica, visando orientar os leitores sobre a doença, origem do estigma associado a ela, papel da APS, sinais e sintomas, diagnóstico, classificação, tratamento e vulnerabilidade.

Além disso, realizou-se uma revisão narrativa de literatura na Bireme e no portal da Coordenação de Aperfeiçoamento de Pessoal de Nível Superior (CAPES), por meio de busca padronizada com as palavras-chave “hanseníase”, “controle da hanseníase”, “hanseníase e APS”.

**Fase 2 - Desenho** - Exigiu uma abordagem multiprofissional entre os pesquisadores, o cartunista e o roteirista. Por motivos didáticos, essa fase foi subdividida em etapas que ocorreram simultaneamente. A montagem da HQ foi organizada nas etapas descritas a seguir.

Etapa de estruturação do roteiro - Decorreu da seleção do conteúdo encontrado na Fase 1 - Análise e adotou a abordagem lúdica, com diálogos cotidianos entre profissional e usuário do serviço de saúde, como estratégia para sensibilizar e capturar a atenção dos leitores.

Etapa de adequação do roteiro - Após a finalização do roteiro pelos pesquisadores, um roteirista especializado em histórias em quadrinhos adequou e corrigiu o texto, conferindo-lhe “vida”.

Etapa de criação de personagens - A seleção dos personagens envolveu a definição das características de cada um, como aparência física, raça/etnia, gênero, profissão e contexto social, visando tornar os personagens mais próximos da realidade cotidiana e fazer com que as histórias remetam a pessoas, ambientes e situações reais do dia a dia.

Etapa de desenho - Para a criação dos desenhos/design, foi utilizado o software Adobe Photoshop. Inicialmente, foram feitos esboços de acordo com o roteiro previamente formatado. Uma vez aprovados, os esboços passaram para a Fase 3, na qual os detalhes de cada cena foram definidos.

Etapa de seleção de hiperlinks - Para fundamentar o conteúdo estabelecido na Fase 1 e promover interatividade nas HQs, optou-se pela inserção de links para leituras complementares sobre o tema apresentado em cada capítulo, quando pertinente.

**Fase 3 - Desenvolvimento** - Foi subdividida em etapas, para melhor prosseguimento.

Etapa de diagramação - Foi realizada em colaboração com o cartunista e com o roteirista. Nesse período, foi definida a quantidade de quadros e balões/falas por página, bem como a dimensão e a linguagem visual (icônica) de cada um deles, organizados da esquerda para a direita^(27)^.

Etapa de colorização - A fase final da construção envolveu a colorização dos desenhos e personagens, após a conclusão de todos os capítulos da história. Com a assistência do cartunista, uma paleta de cores foi determinada para atribuir cores aos quadros e personagens, dando “vida” às histórias.

Em todas as etapas, houve um cuidado especial com a apresentação dos conteúdos, linguagem e estética, sem comprometer o desenvolvimento do conteúdo técnico-científico relacionado ao tema. Esse passo incluiu o uso do pretérito como tempo verbal e de espaçamento simples entre linhas, tanto na apresentação quanto no corpo da história em quadrinhos

**Fase 4 - Implementação** - Utilizou-se como estratégia a ampla divulgação nas principais plataformas de acesso livre da área da enfermagem/saúde, como o Cofenplay. Além disso, foi realizada parceria com a Secretaria Municipal de Saúde de Curitiba para a distribuição da HQ em formato físico nas unidades de saúde da cidade.

Apesar de ser uma história em quadrinhos com personagens imaginários, a narrativa sobre a hanseníase na APS foi cuidadosamente desenvolvida. Buscou-se representar a doença com sensibilidade, evitando estereótipos. Além disso, certificou-se de manter uma base plausível e respeitar as características clínicas, mesmo na ficção. A abordagem incluiu a desmistificação da hanseníase por meio de mensagens esclarecedoras; e a construção de personagens respeitou a diversidade cultural e étnica. A história passou por revisões entre as pesquisadoras, garantindo uma abordagem ética e responsável.

## RESULTADOS

A construção do roteiro levou em conta os conteúdos identificados e atualizados na revisão narrativa e na pesquisa documental. O primeiro esboço da narrativa foi realizado com o desenvolvimento de uma história lúdica e criação de dois personagens centrais: a enfermeira Denise e um jovem curioso chamado Pedro.

Esse roteiro foi retrabalhado por um roteirista de histórias em quadrinhos a fim de dar agilidade e “vida” para a história e personagens. A seguir, a Figura 1 mostra um exemplo de texto após ter recebido intervenção do roteirista.

**Figura 1:** Adaptação do roteiro

Com todas as etapas concluídas, foi decidido que a história em quadrinhos seria dividida em oito capítulos, apresentados a seguir.

Capítulo I. Enigma da Hanseníase - Ilustra a curiosidade de um jovem estudante que, motivado pela necessidade de realizar um trabalho de iniciação científica sobre a hanseníase, depara se com uma série de dúvidas sobre o tema e com a dificuldade de realizar uma pesquisa de boa qualidade, decidindo, então, procurar ajuda de um profissional da saúde.

Esse capítulo destaca a necessidade e a importância da pesquisa e da busca das evidências científicas para construir o conhecimento, seja na formação acadêmica, seja no desenvolvimento profissional.

Capítulo II. Em busca do conhecimento: uma grata surpresa - Evidencia a importância do SUS e dos serviços oferecidos pela APS, retratando a organização estrutural para atendimento dentro de uma unidade de saúde e demonstrando a importância dos enfermeiros enquanto atores para a construção de conhecimento e implementação da educação em saúde.

Capítulo III. Desvendando a história - A intenção desse capítulo é voltar o olhar para a história da hanseníase e sensibilizar o leitor para os aspectos psicossociais associados à doença, ao estigma e às suas consequências, que atravessaram séculos e perduram até hoje. Esses elementos são representados na Figura 2.

**Figura 2:** Layout do capítulo relacionado à história da hanseníase

Capítulo IV. Explorando a doença: sinais e sintomas - O ponto central nesse capítulo é o esclarecimento dos sinais e sintomas da hanseníase, trazendo à tona as principais manifestações e os sinais cardinais, além de levantar a questão da insegurança e do despreparo dos profissionais para realizar o diagnóstico e os cuidados das pessoas acometidas (Figura 3).

**Figura 3:** Layout do capítulo relacionado aos sinais e sintomas

Capítulo V. O diagnóstico - Este capítulo traz um recurso crucial, o diagnóstico, que é considerado por muitos autores um ponto de fragilidade no atendimento às pessoas com suspeita de hanseníase. Ele expõe de forma indubitável e simples quais exames e testes são realizados para a confirmação do diagnóstico, além de orientar sobre o material necessário e suas possíveis substituições^(7)^ (Figura 4).

**Figura 4:** Layout do capítulo relacionado ao diagnóstico

Capítulo VI. Classificação - Enfatiza o esclarecimento e o aprofundamento sobre a classificação da hanseníase, suas formas de manifestação e características principais de cada categoria definida pela classificação de Madrid^(28)^, a saber: a forma paucibacilar está subdividida em forma indeterminada e forma tuberculoide, caracterizadas pela presença de até cinco manchas; já a forma multibacilar é subdividida em forma dimorfa e forma virchowiana, as quais, em geral, apresentam mais de cinco manchas. Os aspectos clínicos e as particularidades de cada uma são revelados de maneira lúdica.

Capítulo VII. Tratamento - Esse capítulo perpassa pela história e pela evolução do tratamento, demonstrando a importância do tratamento com dose supervisionada mensalmente pelos enfermeiros para a cura da doença. Orienta sobre o uso correto e prescrição adequada para cada forma da doença, em consonância com as diretrizes do Ministério da Saúde. Enfatiza o acompanhamento no decorrer do tratamento para evitar o abandono e a notificação obrigatória após a confirmação do diagnóstico (Figura 5).

**Figura 5:** Layout do capítulo relacionado ao tratamento

Capítulo VIII. Vulnerabilidade - O último capítulo ressalta a hanseníase como uma doença tropical negligenciada, o que a coloca em estreita relação com questões de vulnerabilidade. Além disso, evidencia o impacto dessa classificação na vida das pessoas afetadas e a relevância dos estudos realizados para reduzir os efeitos do estigma.

## DISCUSSÃO

A Educação Permanente em Saúde (EPS) desempenha um papel crucial no enfrentamento da lacuna existente na formação profissional, bem como na capacitação dos profissionais de saúde. A EPS, concebida como uma ferramenta educacional, visa superar o despreparo que pode resultar de uma formação focada apenas no desenvolvimento de habilidades técnicas operacionais, sem a integração de conhecimentos multidisciplinares, como aspectos éticos, sociais e políticos^(29,30)^.

Nesse sentido, a construção da história em quadrinhos retrata uma necessidade percebida nos espaços de prática profissional em saúde, onde os profissionais recebem demandas específicas da comunidade atendida, como pacientes com hanseníase, e muitas vezes desconhecem o fluxo de atendimento preconizado pelo Ministério da Saúde para esses casos. Também vale destacar a imprescindibilidade do conhecimento, da expertise, das habilidades e da proatividade dos profissionais de saúde, sobretudo do profissional enfermeiro - primeiro a atender os pacientes com hanseníase na APS - na elaboração e na implementação de estratégias eficazes no reconhecimento precoce das lesões; tais qualidades melhoram o enfrentamento da doença e pavimentam o caminho para sua erradicação no Brasil^(11)^.

Dessa forma, a utilização de HQs destaca-se como uma abordagem eficaz, ao transformar conteúdos complexos em práticas interessantes e motivadoras, uma vez que torna a leitura e a compreensão divertidas, com possibilidade de estimular a aproximação à temática abordada e promover mudanças no processo de ensino-aprendizagem^(27,31)^. Estudos^(32,33)^ corroboram essa eficiência evidenciando o potencial das HQs enquanto ferramenta lúdica da aprendizagem ativa.

Nesse sentido, as Figuras 4 e 5 ilustram a capacidade lúdica das HQs, ao abordar conteúdos desafiadores, como a história da hanseníase e seus sinais e sintomas, com uma linguagem envolvente e mais próxima da prática profissional. A literatura ressalta que o uso de HQs em sala de aula favorece a reflexão sobre novas informações e, consequentemente, contribui para o ganho de conhecimento de maneira complementar aos padrões tradicionais adotados nas instituições de ensino^(34)^.

O capítulo que explora a história da hanseníase e seus estigmas têm como propósito imergir os leitores no contexto histórico e nas experiências vivenciadas, buscando promover uma reflexão profunda sobre a realidade da exclusão social vivida pelas pessoas acometidas. Essa abordagem visa estimular a empatia, enquanto desmistifica os preconceitos e os estigmas ligados a essa doença negligenciada em nosso país. É essencial destacar a relevância dessa abordagem educacional, uma vez que a redução do estigma e do preconceito é um dos princípios fundamentais preconizados pela Organização Mundial da Saúde como caminho para sua erradicação^(2)^.

A inserção da criatividade na construção das HQs desempenha um papel fundamental ao engajar e motivar os profissionais desde o início, como explicitado no “Capítulo I. Enigma da Hanseníase”. A narrativa instigante, centrada na curiosidade de um jovem estudante, é estrategicamente elaborada para capturar a atenção e despertar o interesse pelo tema. Ao introduzir a trama com um enigma, a história estimula a curiosidade do leitor, promovendo um envolvimento ativo na busca por respostas. Essa abordagem não apenas torna a aprendizagem mais atrativa, mas também estabelece uma conexão emocional, visto que a identificação com o protagonista pode criar um vínculo pessoal com a narrativa.

Dessa forma, a criatividade na construção das HQs não é apenas um recurso estilístico, mas também uma estratégia pedagógica eficaz, que visa gerar uma experiência de aprendizado envolvente e memorável. Essa ideia está em consonância com a EPS, que considera o espaço de prática profissional como um dos espaços favoráveis para desenvolver respostas às necessidades dos indivíduos e coletividades^(29)^.

A abordagem visual das HQs exerce uma função central ao oferecer clareza e acessibilidade na apresentação de informações complexas de maneira simplificada. O “Capítulo IV. Explorando a doença: sinais e sintomas” ilustra essa estratégia, concentrando-se na compreensão visual dos aspectos fundamentais da hanseníase^(35)^.

Ao empregar elementos visuais, como ilustrações e diagramas, as HQs proporcionam uma representação gráfica dos conceitos, tornando o conteúdo mais compreensível e memorável. Essa abordagem não apenas atende a diferentes estilos de aprendizado, incluindo aqueles que se beneficiam mais da informação visual, mas também contribui para a eficácia do ensino, pois a visualização pode simplificar conceitos complexos. Dessa forma, a combinação da abordagem visual das HQs com a exploração dos sinais e sintomas da hanseníase no Capítulo IV evidencia a efetividade dessa estratégia na comunicação de informações em saúde de maneira acessível e educativa.

Ao inserir o conhecimento em um contexto prático, a HQ almeja tornar a aprendizagem mais aplicável ao cotidiano dos enfermeiros na APS. O “Capítulo V. O diagnóstico” ilustra de maneira específica essa abordagem ao fornecer informações relevantes diretamente relacionadas à prática diária dos profissionais de saúde. Essa estratégia visa estabelecer uma conexão direta entre os conceitos abordados na HQ e as situações reais enfrentadas pelos enfermeiros na APS, promovendo uma compreensão mais profunda e uma aplicação mais efetiva do conhecimento adquirido.

Esse enfoque demonstra que a consolidação da educação na saúde ocorre por meio da aprendizagem significativa, enfatizando a importância de relacionar o aprendizado teórico com a prática profissional. Assim, a contextualização do conhecimento na rotina dos enfermeiros (Capítulo V), para além de fortalecer a relevância da informação apresentada, contribui para a formação de profissionais mais capacitados para enfrentar desafios do campo da saúde^(36)^.

A HQ busca estimular a reflexão e a empatia ao abordar a história da hanseníase e seus estigmas, a fim de promover uma compreensão mais profunda das experiências dos pacientes. Também visa reforçar a urgência de preparar adequadamente os profissionais que lidam com os pacientes acometidos pela hanseníase, para que reconheçam as lesões precocemente e realizem uma conduta terapêutica oportuna. No “Capítulo VII. Tratamento”, a ênfase ultrapassa os aspectos técnicos, destacando expressamente o papel compassivo do enfermeiro no suporte ao paciente. Além de reconhecer a importância das habilidades técnicas, essa abordagem enfatiza a necessidade de humanização na prestação de cuidados.

Ao propor a superação do paradigma de atendimento e cuidados centrados no modelo biomédico, o enfoque passa a ser nos esforços para implementar uma visão holística e de cuidados integrais às pessoas com hanseníase. Essa mudança de perspectiva reflete a compreensão de que o tratamento não deve se limitar apenas aos aspectos físicos da doença: precisa também abranger as dimensões psicossociais e emocionais, reconhecendo a complexidade das experiências dos pacientes. Para além desses aspectos, deve-se reconhecer que é uma doença negligenciada e, quando não detectada precocemente, é incapacitante, portanto, requer esforços dos profissionais e órgãos de saúde para mitigá-la^(37)^.

Assim, ao incluir o tratamento (Capítulo VII), a HQ fornece informações sobre as práticas médicas e promove uma narrativa cujo fim é sensibilizar os profissionais de saúde e os leitores sobre a importância de uma abordagem compassiva e integral no cuidado àqueles que vivenciam a hanseníase.

Essa abordagem, alinhada com as tendências contemporâneas de cuidados de saúde, destaca a importância de uma visão mais ampla e centrada no paciente para o enfrentamento dessa doença e seus desafios associados.

A natureza multissensorial das HQs, combinando elementos visuais e textuais, visa oferecer uma experiência de aprendizado rica e envolvente. A Figura 3, representando o “Capítulo II. Em busca do conhecimento: grata surpresa”, enriquece a compreensão sobre o ambiente de trabalho dos enfermeiros na APS. Nesse sentido, considera-se que ferramentas criativas para uso na educação permanente, como as HQs desenvolvidas neste estudo, não só atendem à necessidade de atualização profissional: elas também transformam o processo ensino-aprendizagem em uma experiência mais atraente e eficaz para adultos no contexto da saúde^(38)^.

Assim, as HQs configuram-se como uma promissora ferramenta pedagógica, dotada de potencial para integrar conteúdos diversos em um formato acessível e atrativo. Sua linguagem visual e narrativa favorece a criação de ambientes lúdicos de aprendizagem, promovendo o diálogo, a troca de saberes e o fortalecimento das dimensões social e imaginativa dos sujeitos^(35,39)^.

Apesar dessas potencialidades, o uso das HQs ainda não é amplamente consolidado no âmbito educacional, particularmente na área da saúde; portanto, são necessárias investigações mais aprofundadas evidenciando sua eficácia e aplicabilidade. Entretanto, observa-se um esforço institucional, sobretudo do Ministério da Saúde, para incorporá-las a iniciativas educativas. Tal movimento justifica-se pela capacidade desse recurso de abordar uma ampla gama de temas e de alcançar diferentes segmentos populacionais - incluindo grupos em situação de vulnerabilidade - por meio de estratégias comunicativas inclusivas e contextualizadas^(35,38,39)^.

### Limitações do estudo

As limitações desta pesquisa incluem a necessidade de validar o produto desenvolvido, validar o constructo e a aparência, bem como avaliar seu impacto no processo de EPS. A validação do produto é um passo decisivo para assegurar a eficácia e a adequação do material educativo, garantindo que atenda aos objetivos propostos. Esse processo envolve a revisão e a avaliação por especialistas na área, educadores e, possivelmente, o público-alvo, a fim de verificar a qualidade, a precisão e a relevância do conteúdo das histórias em quadrinhos.

Outra limitação a ser considerada é a necessidade de avaliar o impacto do material no processo de EPS. Embora as HQs tenham potencial para serem ferramentas eficazes de aprendizado, é fundamental medir como essas intervenções influenciam a formação contínua dos profissionais de saúde. A avaliação do impacto pode incluir indicadores como mudanças no conhecimento, atitudes e práticas dos profissionais, assim como a sua percepção sobre a utilidade e relevância das HQs no contexto da EPS.

Além disso, a generalização dos resultados é limitada, pois a pesquisa foi desenvolvida em um cenário particular e com um perfil específico de profissionais de saúde. Portanto, a replicação do estudo em diferentes contextos e com amostras mais diversas é recomendada para validar a aplicabilidade e eficácia das HQs como ferramenta de EPS em variadas situações de saúde e entre distintos perfis profissionais.

Considerando a importância da validação e da avaliação do material educativo, optou-se por analisar o impacto da história em quadrinhos após sua divulgação na versão impressa, realizada pela parceria entre a secretaria municipal de saúde e a secretaria de educação de um município da Região Metropolitana de Curitiba.

A avaliação será conduzida por meio de um questionário estruturado na plataforma *Google Forms*, seguindo um roteiro preestabelecido para analisar os seguintes aspectos: *design* e clareza do texto, criatividade e originalidade, eficácia educacional e adequação ao público-alvo. O instrumento utilizará questões em escala Likert combinadas com questões abertas para capturar aspectos subjetivos relacionados à experiência individual de cada leitor.

### Contribuições para a área da enfermagem, saúde ou política pública

Nesse cenário, os quadrinhos emergem como uma ferramenta educacional de grande valor, capaz não apenas de estimular a criatividade dos alunos, mas também de fortalecer as interações entre eles. Essa perspectiva é respaldada por um estudo recente que examinou o uso de histórias em quadrinhos na educação física, visando promover a saúde no ensino fundamental brasileiro^(39)^.

Para além de seu potencial educativo, as HQs também têm como objetivo alcançar outros propósitos significativos. A natureza lúdica dessas narrativas gráficas pode fornecer uma contribuição determinante na redução dos níveis de estresse e ansiedade, tanto para os pacientes quanto para os profissionais de saúde. A leitura de HQs emerge como uma ferramenta valiosa para auxiliar os trabalhadores da saúde e a comunidade em geral a superar experiências desafiadoras, ao mesmo tempo que estimula a empatia por meio das histórias compartilhadas por outras pessoas^(40,41)^.

Destaca-se que o trabalho de interação multiprofissional foi fundamental para o desenvolvimento do estudo, haja vista a necessidade de adaptação do conteúdo técnico-científico proposto pelos autores no roteiro inicial, as características do material e as exigências das técnicas para a construção de histórias em quadrinhos.

## CONSIDERAÇÕES FINAIS

A pesquisa destaca a significativa contribuição das HQs como ferramenta educacional para fortalecer a formação profissional em saúde, particularmente no contexto da hanseníase, já que se trata de uma doença negligenciada no Brasil. Assim, demonstrou-se que sua utilização não somente enriquece o processo de ensino-aprendizagem, tornando temas complexos mais acessíveis e envolventes, como também promove a criatividade dos alunos e fortalece interações sociais.

Além de apontar o potencial das HQs em abordar aspectos educativos, os resultados também evidenciam uma contribuição para a superação de desafios emocionais, reduzindo níveis de estresse e ansiedade em pacientes e profissionais de saúde. Paralelamente, a ferramenta estimula a empatia por meio de narrativas compartilhadas, ou seja, fortalece o conhecimento técnico-científico dos profissionais de saúde da APS para a atenção à hanseníase, enquanto promove uma abordagem criativa da temática.

Entretanto, é fundamental reconhecer as limitações da pesquisa. Os resultados referem-se a um contexto específico, o que limita sua generalização; logo, é necessário replicar o estudo em diferentes cenários e com amostras mais diversas para validar a aplicabilidade e a eficácia das HQs como ferramenta de EPS em variadas situações de saúde e entre distintos perfis profissionais.

## Data Availability

Os dados de pesquisa estão disponíveis em repositório: https://doi.org/10.48331/scielodata.IT9VIP.
